# Efficacy and Satisfaction Rate in Postpartum Intrauterine Contraceptive Device Insertion: A Prospective Study

**DOI:** 10.7759/cureus.5646

**Published:** 2019-09-13

**Authors:** Pulwasha M Iftikhar, Nighat Shaheen, Ena Arora

**Affiliations:** 1 Obstetrics and Gynecology, St. John's University, New York, USA; 2 Obstetrics and Gynecology, Cantonment General Hospital, Rawalpindi, PAK; 3 Obstetrics and Gynecology, Icahn School of Medicine, Mount Sinai, New York, USA

**Keywords:** postpartum intrauterine contraceptive device, long-acting reversible contraception, family planning, planned pregnancy, satisfaction rate

## Abstract

Background

Postpartum intrauterine contraceptive device (PPIUCD) reduces the rate of abortions and it is a cost-effective, reversible, and convenient choice of contraception. The objective of our study was to evaluate the efficacy and satisfaction rate in women with postpartum intrauterine contraceptive device insertion.

Methods

This prospective study of immediate PPIUCD insertion was conducted at our institute from March 2016 to February 2019. Approval from the Institutional Review Board (IRB) was taken before starting the study. A total of 372 women were enrolled in the study after taking informed consent. All the women were counseled regarding different methods of contraception and birth control during antenatal checkups, early labor and immediately postpartum (within 48 hours). All the enrolled women in the study were followed for three years to determine the satisfaction and success rate of PPIUCD continuation. We also kept the record of women who discontinued PPIUCD.

Results

After the exclusion criteria, 372 women were recruited in the study. The mean gestation age at the time of delivery was 38.5 weeks with a standard deviation (SD) of 1.45. All the women were followed for short-term and long-term complications and satisfaction rates. Out of 372, 51.07% of women (n = 190) had a spontaneous vaginal delivery, and 48.9% of women (n = 182) had a cesarean section but there was no significant long-term satisfaction outcome difference in both the groups. The highest success rate of the postpartum long-acting intrauterine contraceptive device was noted in patients who were counseled thoroughly in the antenatal and intrapartum period 61.5% as compared to those patients who were counseled either antenatally 42.2 %, intrapartum 35.4%, or immediate postpartum 22.4% alone.

Conclusion

PPIUCD insertion is an opportunity not to be missed. It allows women to obtain safe, long-acting, highly effective contraception while already within the medical system. More research data are needed in the literature with regard to counseling timings for PPIUCD insertion during the antenatal and postnatal period as it can affect the decision of women to prevent unplanned pregnancy. PPIUCD has one of the highest patient satisfaction rates among all the contraceptives.

## Introduction

Postpartum family planning is the prevention of unintended and closely spaced pregnancies during the first 12 months following childbirth [1]. Unintended pregnancy is characterized by untimely and short pregnancy intervals and it can result in acute maternal complications and death of mothers and their children. In the United States, half of the pregnancies are unintended. According to “Healthy people 2020,” almost 6.1 million pregnancies are unplanned and it has a direct association with negative health and economic outcomes [2]. An unplanned pregnancy can cause maternal and child morbidity and mortality. In a recent study of postpartum unintended pregnancies, 86% resulted from non-use of contraception and almost 50% ended in induced abortions [2-4]. Using family planning (FP) to space births at least 36 months apart can avert 30% of maternal deaths and 10% of child deaths [3-4]. 

Insertion of an intrauterine contraceptive device (IUCD) immediately after delivery has been recommended by the World Health Organization (WHO), as one of the safe and effective methods of temporary contraception [5]. Postpartum intracontraceptive uterine device (PPIUCD) can be safely used in all breastfeeding women. Almost 39% to 65% of women in the first-year postpartum have an unmet need for family planning [5-7]. Hence, providing contraception in this sensitive period is important. PPIUCD reduces the rate of abortions and it is a cost-effective, reversible, and convenient choice of contraception.

Early resumption of sexual activity coupled with early and unpredictable ovulation leads to many unwanted pregnancies in the first year postpartum. Moreover, in developing countries particularly, women who once go back home after delivery do not return for even a routine postpartum check-up, leave aside contraception. There are many reasons for not using contraception, specifically long-acting reversible contraception (LARC), including lack of awareness, non-availability of accessible family planning services, social pressure, and limitations on women’s mobility mostly due to cultural or geographical factors. Thus, immediate postpartum family planning services need to be emphasized wherein the woman leaves the hospital with effective contraception in place. Institutional deliveries create a unique opportunity to offer a long-acting yet reversible method of contraception to women immediately following their childbirth. Cochrane reviews provide evidence of the safety and feasibility of Long-acting reversible contraception insertions in various settings [8-9].

Initiation of family planning at the time of birth is opportune, since few women in low-resource settings who give birth in a facility return for further care [7-8,10]. PPIUCD is an effective long-acting reversible contraceptive, which encourages women to give birth in health care facilities [11-12]. LARC has several advantages for use in the postpartum period as it is effective, coitus independent and does not interfere with breastfeeding.

Effective intervention is required at an individual, interpersonal, community and institutional levels, and policymakers should play their role in providing affordable and easily accessible methods of contraceptive to improve maternal and child health. Provision of awareness and education about different methods of contraception during the antenatal and postnatal period reduce the rate of unplanned pregnancies. Healthcare professionals should be well trained for the appropriate insertion of IUCD in the postpartum period [8,10-11].

## Materials and methods

This prospective study of immediate PPIUCD insertion was conducted at our institute from March 2016 to February 2019. Approval from the Institutional Review Board (IRB) was taken before starting the study. A total of 372 women were enrolled in the study after taking informed consent. All the women were counseled regarding different methods of contraception and birth control during antenatal checkups, early labor and immediately postpartum (within 48 hours). In the enrolled patients, IUCD was inserted immediately postpartum regardless of the mode of delivery either intra-cesarean section (CS) or spontaneous vaginal delivery (SVD). Some patients took longer to make the decision, and IUCD was inserted after vaginal delivery within 48 hours. All the patients were followed up at six weeks, six months, and yearly for consecutive three years.

Exclusion criteria for IUCD included uterine abnormalities (unicornuate, bicornuate, didelphus, or septate uterus), hemoglobin (Hb) of less than 8 g/dl, coagulation disorders, fever and infection during labor, prolong rupture of membranes ≥18 hours, active lower genital tract infection and sexually transmitted diseases (STD), postpartum hemorrhage and manually removing the placenta.

Questionnaire about secondary postpartum hemorrhage (PPH), infection, expelled IUCD before follow-up, displaced IUD, and short- and long-term satisfaction rate was filled out. All the enrolled women in the study were followed for three years to determine the satisfaction and success rate of PPIUCD continuation. We also kept the record of women who discontinued PPIUCD. 

All the data were statistically analyzed on SPSS version 25.0. Chi-square, Student *t*-test, and Mann-Whitney U test were used for categorical variables. For the determination of factors that affected success, logistic regression was used.

## Results

**Figure 1:**
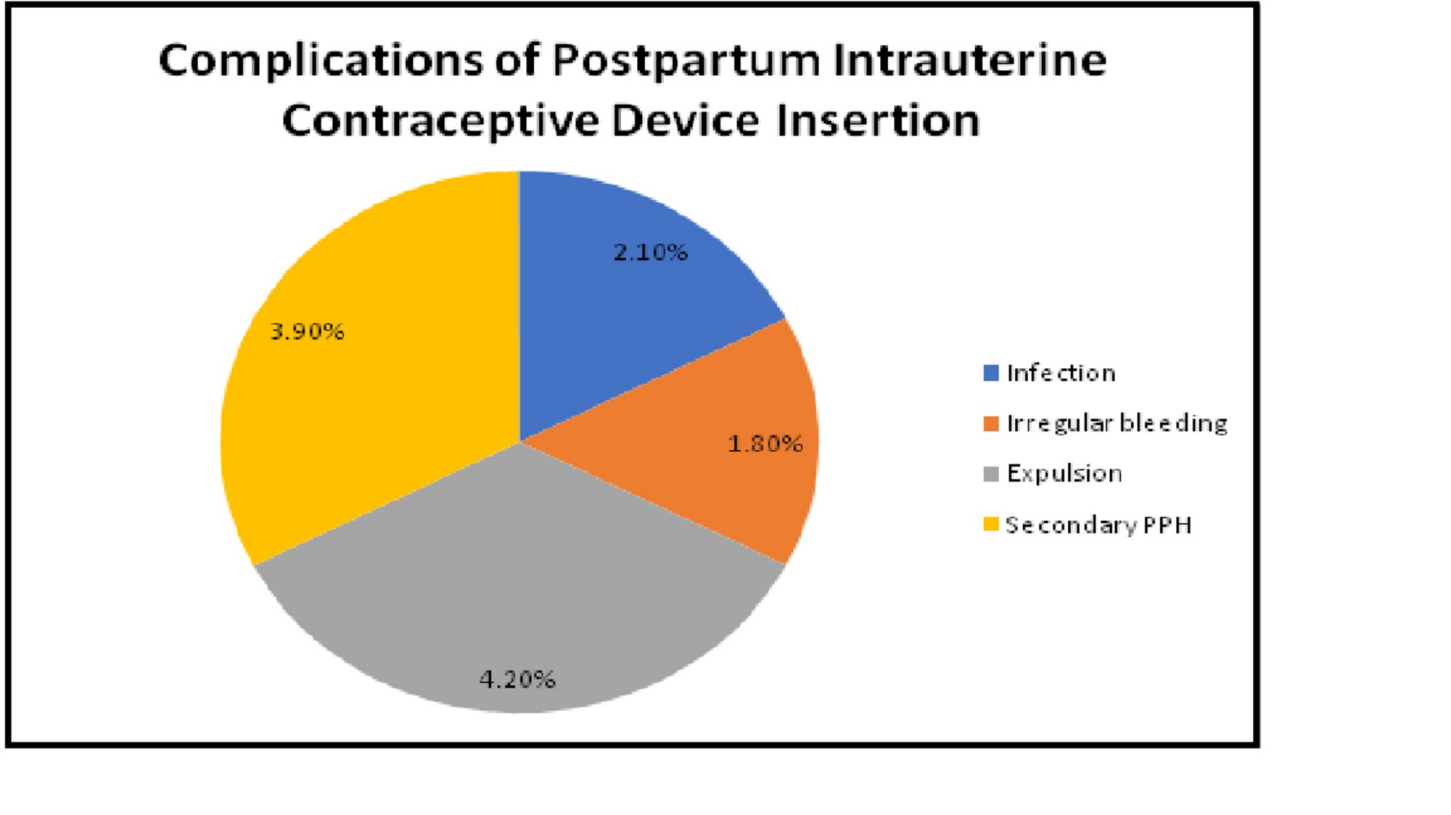
Complications of postpartum intrauterine contraceptive device PPH, postpartum hemorrhage

After the exclusion criteria, 372 women were recruited in the study. The mean gestation age at the time of delivery was 38.5 weeks with a standard deviation (SD) of 1.45. All the women were followed for short-term and long-term complications and satisfaction rates. Out of 372, 51.07% of women (n = 190) had a spontaneous vaginal delivery, and 48.9% of women (n = 182) had a cesarean section, but there was no significant long-term satisfaction outcome difference in both the groups. Pelvic ultrasound was performed on each visit to ensure that IUCD is in situ. On six weeks postpartum visit, 2.9% women (n = 11) requested PPIUCD removal due to dissatisfaction and abdominal discomfort. Other short-term complications included irregular bleeding in 1.8% (n = 6), secondary PPH was 3.9% (n = 14), and infection in 2.1% (n = 7). PPIUCD was expelled in 4.2% patients (n = 15) and displaced in 0.005% patients (n = 2; Figure 1). However, there was no reported case of uterine perforation in this study. On subsequent visits, 2.1% (n = 8) women requested IUCD removal due to interest in conception, menstrual irregularities, sexual discomfort, and abdominal cramping.

The highest satisfaction rate was observed in multiparous women between the ages of 20 to 25 ± 5 years. Overall, the satisfaction rate was 97.2 % (n = 350) at the time of insertion and 95% on the subsequent six weeks postpartum visit. Further, follow-up visits at six months and yearly for consecutive three years showed an average satisfaction rate of 93.95% (Table 1). 

**Table 1:** Satisfaction rate of postpartum intrauterine contraceptive device

Three-year Satisfaction Rate of Postpartum Intrauterine Contraceptive Device Insertion		
1	Satisfaction rate at time of insertion	97.2%
2	Satisfaction rate at six-weeks postpartum	95%
3	Satisfaction rate at six-months postpartum	94.5%
4	Satisfaction rate at one-year postpartum	93%
5	Satisfaction rate at two-years postpartum	93%
6	Satisfaction rate at three-years postpartum	91%
7	Overall satisfaction rate	93.95%

The highest success rate of the postpartum long-acting intrauterine contraceptive device was noted in patients who were counseled thoroughly in the antenatal and intrapartum period 61.5% as compared to those patients who were counseled either antenatally 42.2%, intrapartum 35.4%, or immediate postpartum 22.4% alone.

## Discussion

LARC to prevent pregnancy is among the oldest and safest methods of contraception. The modern IUCD is a highly effective, safe, private, long-acting, coitus independent and rapidly reversible method of contraception with fewer side effects. Nowadays, IUCD is the most cost-effective method of contraception and many women also find it to be very convenient because it requires little attention once inserted [3,5,8]. The intrauterine contraceptive device is considered one of the most reliable, inexpensive, non-hormonal and reversible contraceptive methods suitable for a lactating mother because it has no negative effects on lactation and may increase its duration in some women and do not affect the quality of the breast milk.

Across the world, particularly in developing countries, the use of long-acting reversible forms of contraceptive methods, especially PPIUCD, is being promoted largely in the postpartum period. Most women are sexually active by six weeks postpartum, and women who deliver by cesarean section may be more likely to resume sexual activity earlier than women who had vaginal deliveries [12-14]. The postpartum period is potentially an ideal time to begin contraception as women are more strongly motivated to do so at this time, which also has the advantage of being convenient for both patients and health-care providers. Other advantages of insertion of an IUCD after delivery are that the discomfort related to interval insertion can be avoided and any bleeding from insertion will be disguised by lochia [15-17].

A study by Shukla et al. reported 27.2% of women had menorrhagia after PPIUCD insertion [16]. However, in this study, the rate of secondary PPH was reported as 3.9%. Welkovic et al. studied postpartum bleeding and infection after post placental IUCD insertion and found no difference in the incidence of excessive bleeding and infection [18].

This study was conducted to evaluate the safety and efficacy of immediate PPIUCD insertion in women delivering vaginally or by cesarean section. A total of 372 women were evaluated. The expulsion rate was found to be 4.2%, which is low as compared to the other studies. From Turkey, Celen et al. reported an 11.3% cumulative expulsion rate for postpartum insertion of CuT 300B [19]. Thiery et al. from Belgium have reported a 9.4% expulsion rate at six months for immediate post-placental insertion. The timing of insertion, counseling, and provider training are important factors for IUCD insertion in the postpartum period as quoted in the United Nations Population Information Network (UNPOPIN) report. Of these, the timing of insertion is important as it influences the risk of expulsion [20]. 

A research conducted in 13 countries studied infection (PID) due to IUCD and they have reported a 2.4% of infection with immediate insertion and interval insertion, which was similar to the rate of infection in our study (2.1%). A study conducted by Ross and Winfrey in 27 countries showed that there were 65% of women who wanted to postpone the next pregnancy but they were not using any method of contraception. However, 39% of women who had delivery in the last one year had an unmet need for contraception [8-9,12,17,21].

Overall, the satisfaction rate was 97.2% at the time of insertion and 95% on the subsequent six weeks postpartum visit. By delaying birth control initiation until the six-week postpartum visit, the most vulnerable women, those who are least likely to return for their six-week postpartum visit are being placed at an increased risk for unintended pregnancies and a short pregnancy interval [16-18].

Postpartum IUCD insertion is an opportunity not to be missed and delivery may be the only time when a healthy woman comes into contact with health care providers and the chances of returning for contraceptive advice are uncertain. It allows women to obtain safe, long-acting, highly effective contraception while already within the medical system. Nowadays, PPIUCD has been established as an effective and reliable method of contraception as it offers numerous advantages: ease of insertion, minimal adverse impacts on breast-feeding, cost-effectiveness, relief of overcrowded outpatient facilities, and protection against unwanted pregnancy and consequent abortion [13-14,19].

## Conclusions

PPIUCD insertion is an opportunity not to be missed. It allows women to obtain safe, long-acting, highly effective contraception while already within the medical system. Nowadays, PPIUCD has been established as an effective and reliable method of contraception as it offers numerous advantages: ease of insertion, minimal adverse impacts on breast-feeding, cost-effectiveness, relief of overcrowded outpatient facilities and protection against unwanted pregnancy and consequent abortion. More research data is required in the literature with regards to counseling timings for PPIUCD insertion during the antenatal and postnatal period as it can affect the decision of women to prevent unplanned pregnancy. PPIUCD has one of the highest patient satisfaction rates among all the contraceptives.
